# The use of complementary and alternative medicine for patients with traumatic brain injury in Taiwan

**DOI:** 10.1186/1472-6882-12-211

**Published:** 2012-11-06

**Authors:** Bih-Shya Gau, Hsiao-Ling Yang, Sheng-Jean Huang, Meei-Fang Lou

**Affiliations:** 1 Department of Nursing, College of Medicine, National Taiwan University, Taipei, Taiwan; 2 Department of Medicine, College of Medicine, National Taiwan University, Taipei, Taiwan; 3 Department of Surgery, National Taiwan University Hospital, Taipei, Taiwan

**Keywords:** Complementary and alternative medicine (CAM), Belief in complementary and alternative medicine, Traumatic brain injury (TBI)

## Abstract

**Background:**

The use of complementary and alternative medicine (CAM) continues to increase in Taiwan. This study examined the use of CAM and beliefs about CAM as expressed by patients with traumatic brain injury (TBI) in Taiwan.

**Methods:**

TBI patients and their accompanying relatives were interviewed by using a structured questionnaire at an outpatient clinic in a medical center in northern Taiwan.

**Results:**

A total of 101 patients with TBI participated in the study. Sixty-four (63%) patients had used at least one form of CAM after sustaining TBI. CAM users had used an average of 2.72 forms of CAM after sustaining TBI. The most frequently used CAM category was traditional Chinese medicine (37; 57.8%), followed by folk and religious therapies (30; 46.9%), and dietary supplements (30; 46.9%). The majority of the patients (45; 70.3%) did not report CAM use because they felt it was unnecessary to do so. Patients who used CAM had a significantly stronger positive belief in CAM than those who did not (t = −2.72; *P* = .008). After using CAM, most of the patients (54; 85%) perceived moderate satisfaction (2.89 ± 0.44), according to a 4-point Likert scale.

**Conclusion:**

Although the use of CAM is common for TBI patients receiving conventional medical health care in Taiwan, most patients did not inform health care personnel about their CAM use. TBI patients perceive combined use of CAM and conventional medicine as beneficial for their overall health.

## Background

Complementary and alternative medicine (CAM) is a medical and health care modality that exists outside conventional medicine and includes many philosophical beliefs and health approaches that are not part of conventional medicine. Combining Chinese medicine with conventional medicine, as well as and the use of folk therapies, is a common practice in Taiwanese culture [1]. CAM has been gradually accepted as a helpful addition to conventional medicine. CAM use is widespread in Taiwan and over 70% of the public has reported using different forms of CAM such as Chinese herbs and folk therapies [2,3]. CAM has mostly been used in Taiwan to improve overall health status, to treat diseases, and to improve quality of life [3].

There is a growing interest in CAM use among patients with chronic conditions. A prevalence of 17–72.3% CAM use has been reported among diabetic patients. Nutritional supplements and herbal medicines were the most frequently used CAM [4]. Chen surveyed outpatients from a hospital in the Taipei area, exploring the prevalence of combining CAM with conventional medicine. The result indicated that 25.6% of the patients used CAM in combination with conventional medicines [1]. Studies from Japan and the United States have also indicated a prevalence of 76% and 38%, respectively for CAM use [5,6]. Most patients do not inform their doctors about using CAM, mostly because they were not asked when reporting medical history [2,3,7].

The characteristics of CAM users differ according to culture, geography, and type of dominant health care system. In surveying the public by telephone in Taiwan, Ting [2] found that CAM use was not associated with demographic variables such as age, gender, educational level, or place of residence. However, a survey by Huang et al. showed that most CAM users were women aged 20 to 49 years who had religious beliefs and educational levels higher than junior college [3]. Harris et al. reported that CAM use after an illness was associated with being female, a young age, a high educational level, and CAM use before an illness [8]. In Turkey, Ceylan et al. examined CAM use among cancer patients and discovered that CAM use was associated with country of birth, low educational level, and extended family structure [9].

A review of relevant literature showed that most recent studies have focused on CAM use in the general population [2,3] or on patients with chronic conditions [4,10]. Some researchers have used quantitative methods to explore the prevalence, effects, and costs of using CAM [3,8,9], whereas others have used qualitative research methods to determine the reasons for CAM use [11,12]. There is a dearth of information about the use of CAM for patients with TBI, a condition that usually occurs following a head injury after sustaining an accident. TBI can lead to varying levels of impaired physical mobility, emotional and behavioral problems, and memory loss. Some of these functions can be recovered, although some patients suffer long-term sequelae. Based on clinical observation, patients with TBI or their families seek conventional medical treatment after the accident, and afterward use diverse complementary therapies to assist in recovery from TBI when conventional medicine is unable to restore impaired physical mobility and overcome long-term sequelae.

### Objectives

This study examined the use of CAM and beliefs about CAM as expressed by patients with traumatic brain injury (TBI) in Taiwan.

## Methods

### Participants and setting

We recruited a convenience sample of adult patients who were diagnosed with TBI and admitted to the neurosurgery ward of a medical center and who, regardless of whether they were to undergo surgery or not, had to continue follow-up after discharge from the TBI outpatient clinic of the study hospital. The TBI outpatient clinic was for TBI patients only. The majority of patients in this outpatient clinic were patients who were previously admitted, managed and discharged from the study hospital. The rest of them were TBI patients who were treated at other hospitals but chose to follow-up at this clinic because of location or being self-referred. Those patients who were not admitted to the hospital during acute stages were excluded from this study. Depending on the conditions of the patients, the duration and frequency of follow-up varied. The average frequency for follow-up was every two weeks to every month.

### Design

This study used a cross-sectional interview-based questionnaire survey to collect information. Data were collected in an outpatient clinic for brain-injury patients in a medical center in northern Taiwan.

### Questionnaire design

The questionnaire included the following three parts:

#### Demographic information

Demographic information included gender, age, marital status, education level, occupation, and living situation. In addition, we assessed the patient’s perceived satisfaction with the overall effects of CAM using a four-point Likert scale ranging from 1 (*very unsatisfied*) to 4 (*very satisfied*).

#### Use of CAM after brain injury questionnaire

The investigators designed this part of the questionnaire based on relevant literature [10,13] and clinical experience. Five categories of CAM with 30 CAM forms were addressed: (1) traditional Chinese medicine such as Chinese herbs and acupuncture; (2) folk and religious medicine, such as holy water, reciting a Buddhist mantra, or spiritual healing techniques (such as shamanism and dang-kis); (3) dietary supplements, such as an organic diet, megadose vitamins, and green tea powder; (4) herbal and arcanum therapy; and (5) mind and body medicine, such as meditation, qi-gong, and aromatherapy. The questionnaire assessed the number of therapies employed by the patients, the reasons for using CAM, patients’ satisfaction with the effects of CAM, and whether the patient discussed CAM with a physician before using it. Three experts in the field, using the same Likert scale, evaluated the relevance and clarity of the content of each question. The inter-rater assessment agreement Kappa was 0.63 (P < .01).

#### Belief in conventional medicine and CAM questionnaire

This part of the questionnaire was adapted from the work of Huang et al. [14]. The participants were asked to rate their agreement with 13 descriptors, such as “scientific,” “less side effects,” and “cured” for CAM and for conventional medicine on a 5-point Likert scale, 1 (*strongly disagree*) to 5 (*strongly agree*), in which a higher score reflected a more positive belief regarding CAM or conventional medicine. Score totals ranged from 13 to 65. The internal consistency (Cronbach’s α) of this questionnaire was 0.90.

### Data collection

A trained research assistant (SPL) collected the data. After explaining the aims of the study and its procedures and before conducting interviews, the patients and family caregivers gave their written consent. Face-to-face interviews were conducted in the waiting area of the brain-injury outpatient clinic. To reduce the recall bias, the information was mainly obtained from the patient, but the accompanying relatives provided additional information to complete the interview data. If the patient had language or cognitive impairment, then the accompanying family caregiver answered as a proxy.

### Data analysis

After coding the data, we used the social sciences statistics program SPSS 16.0 for Windows (Chicago, USA) for analysis. We employed descriptive and inferential statistics in accordance with the aims of this study, and α = .05 was considered to have statistical significance.

### Ethical considerations

This study was reviewed and approved by the Research Ethics Committee of National Taiwan University Hospital (No. 200612054R), where it took place. Participants gave written consent. They were informed that the data produced in this study would be confidential, would not affect patient treatment, and would be used only for academic research.

## Results

### Demographic information on participants

A total of 101 patients with TBI were interviewed, 91 of whom were conscious. The information was obtained mainly from the patients; however, for the 10 patients with altered level of consciousness, the accompanying relatives provided information. Most of the patients were women (62; 61.4%), unemployed (63; 62.4%), married (56; 55.4%), and were living with families (94; 93.1%). Their mean age was 45 years (S.D. = 18.86), mean year of education was 11 years (S.D. = 4.39), and mean period of head injury before being interviewed was 28 months (S.D. = 32.48). The mean score for perceived satisfaction with the overall effects of CAM was 2.89 ± 0.44 (range: 2–4), indicating a level slightly below “satisfied” (Table 1).

**Table 1:** Demographic information of participants (n = 101)

**Variable**	**Category**	**n**	**%**	**Mean ± SD (Range)**
Age (year)				45.48 ± 18.86
				(20–98)
Gender	Male	39	38.6	
	Female	62	61.4	
Education (years)				11.03±4.39
				(0–18)
Occupation	None	63	62.4	
	Employed full-time	23	22.8	
	Employed part-time	6	5.9	
	Student	9	8.9	
Marital status	Single	38	37.6	
	Married	56	55.4	
	Divorced	3	3.0	
	Widowed	4	4.0	
Living situation	With family	94	93.1	
	Alone	7	6.9	
Perceived effects				2.89 ± 0.44
				(2–4)

*SD*: standard deviation.

### The use of CAM for TBI patients

Most patients (86; 85.1%) had never used any form of CAM therapy prior to their brain injury. Sixty-four (63%) patients had used at least one form of CAM after sustaining TBI. The mean time from injury to beginning use of CAM was 1.7 months (S.D. = 2.27). An average of 2.72 (range: 1–12) CAM forms was used after TBI. The most frequently used CAM category was traditional Chinese medicine (37; 57.8%), followed by folk and religious therapies (30; 46.9%), and dietary supplements (30; 46.9%) (Table 2). The main reasons for using CAM were to improve overall health (26; 40.6%), as complementary to conventional medicine to restore health (26; 40.6%), and because they were suggested by relatives and friends (25; 39.1%) (Table 3). The majority of the patients (45; 70.3%) did not report CAM use because they felt it was unnecessary to do so. However, the majority (59; 92.2%) continued using conventional medicine (Table 4).

**Table 2:** The use of CAM by traumatic brain injury patients (n = 101)

**Variable**	**n**	**%**	**Mean ± SD (Range)**
Use of CAM following TBI			
Yes	64	63.4	
No	37	36.6	
Time after injury until beginning use of CAM (months)			1.73±2.27
			(0–12)
Most commonly used CAM category^a^			
Traditional Chinese medicine	37	57.8	
Folk and religious therapy	30	46.9	
Dietary supplements	30	46.9	
Mind-body medicine	7	10.9	
Herbal and Arcanum therapy	6	9.4	
Most commonly used CAM form^a^			
Chinese herbs	30	46.9	
Spiritual healing, such as shamanism, dang-kis	21	32.8	
Special diets	17	26.6	
Megadose vitamins	16	25.0	
Holy water, reciting a Buddhist mantra	15	23.4	
Acupuncture	14	21.9	

*CAM*: complementary and alternative medicine, TBI: traumatic brain injury

*SD*: standard deviation.

^a^: Data from those who used CAM (N = 64).

**Table 3:** Reasons for using CAM after brain injury (n = 64)

**Reason**	**Frequency**	**%**
To improve overall health	26	40.6
As a complement to conventional medical treatment	26	40.6
Suggestion by relatives and friends	25	39.1
For psychological and spiritual support	19	29.7
To relieve symptoms	18	28.1
To rebuild confidence in ability to overcome disease	10	15.6
To alleviate stress	9	14.0
No progress in current condition with conventional medicine	3	4.7

*CAM*: complementary and alternative medicine.

**Table 4:** Attitudes of CAM users toward conventional medicine (n = 64)

**Item**	**n**	**%**
Discussed CAM with physician		
No	50	78.1
Yes	14	21.9
Reasons for concealing the fact of using CAM^a^		
Not necessary to tell	45	90.0
Afraid the physician may not be happy if she/he knows it	2	4.0
To avoid distracting the physician	1	2.0
Afraid the physician will prohibit using CAM	1	2.0
Physician is too busy to talk on the topic	1	2.0
Discontinuing the conventional medicine regimen		
Yes	5	7.8
No	59	92.2

*CAM*: complementary and alternative medicine.

^a^: Data from those who used CAM but did not discuss it with a physician (N = 50).

### Belief in conventional medicine and CAM

The total score for belief in the effectiveness of CAM (31.09 ± 16.38; range14-64) was lower than the belief in conventional medicine (51.42 ± 5.35; range: 40–65). There were statistically significant differences (t = −2.72; *P* = .008) in the score of belief in CAM between patients who used CAM (34.36 ± 15.11) and those who did not use CAM (25.43 ± 17.15) after brain injury, indicating that the patients who used CAM had a significantly stronger positive belief in CAM than those who did not.

## Discussion

A prevalence of 63.4% for CAM use by patients with TBI was obtained in this study. This was similar to the rates reported for cancer patients in Turkey [9], and pediatric cancer patients in Taiwan [10]. The prevalence rate was lower than the rates reported for the public in Japan [5] and cancer patients in Taiwan [13] but higher than those for cancer patients in the United States [7] and the United Kingdom [8]. Differences in the definition of CAM and study population accounted for the wide variation of rates in the results of this study. Chinese medicine was the most frequently used CAM in this study. This is contrasting to the types of CAM use in other studies [3,7,8]. This might have resulted from cultural differences and the convenience of use.

More than 90% of the patients continued using conventional medicine while using CAM. This is a typical Taiwanese pattern of mixing traditional Chinese medicine, conventional medicine, and folk therapies [15]. Similar findings have been reported in many developing countries [16,17]. Medical practices in Taiwan are generally categorized into three groups: conventional medicine, traditional Chinese medicine, and folk medicine. Each type of medicine consists of specific knowledge, skills and beliefs, and diverse approaches that coexist, in accordance to the definition of a “mixed” medical system or medical pluralism. Medical pluralism, which is the combination of conventional and traditional medical systems, is widespread in Asian countries. In many countries, the public believes that simultaneously applying CAM with conventional treatments improves overall health, which is consistent with beliefs regarding health and illness [15,18]. Chinese culture understands health regarding the concepts of yin and yang, emphasizing harmony and balance. This includes internal harmony of the body and harmony between the body and its environment. For example, food may be used as medicine to improve the internal harmony of the body [15].

In our study, patients with TBI used CAM primarily to improve their overall health and, additionally, complement conventional medical treatment to restore health. Thus, TBI patients have used CAM mainly to preserve their health with the hope of strengthening the body, which is similar to findings from a study of the public in Taiwan [19]. Another major reason for using CAM was for psychological and spiritual support and to rebuild confidence in the ability of the patients to overcome illness. Half of the participants in our study believed that spiritual healing, the use of holy water, and reciting a Buddhist mantra strengthen the psychological domain, which reflects the concept that body and mind affect each other. Such psychological support can assist patients in coping with acute conditions or the long-term problems they must confront every day. Moreover, spiritual therapies may help people cope with the exigencies of daily life, as well as with special problems.

Most participants in this study (78.1%) did not inform their physicians on their CAM use, a finding that is similar to those of previous studies [3,19,20]. For example, in Taiwan, Tseng et al. [19] and Huang et al. [3] found that 81.6% and 50% of CAM users, respectively, did not inform conventional health care personnel. Cheung et al. [20] reported that reasons for not disclosing CAM use included “not being asked,” “didn’t think about it,” and “didn’t think it was important to my care.” However, not informing physicians on CAM use may increase the risk of health complications related to combined use of CAM and conventional medicine. Physicians must know enough about various forms of CAM to know which should be contraindicated with the treatments they are providing and which need not be. This reduces the risk of CAM modality in adversely affecting treatment plans designed by physicians.

Characteristics of CAM users differ according to culture, geography, and type of dominant health care system. Our study showed that CAM use in Taiwan is not associated with variables such as patient gender, educational level, or religious beliefs, and whether patients had used CAM in the past, which is similar to the results of most studies [8,9,13,19]. We found that younger patients who believe in CAM were more likely to use CAM. This was similar to the findings from other studies that reported a relationship between age and CAM use [3,4,21]. Previous studies have shown that the response “had ever used CAM” was a critical predictive variable for current CAM use [1,8]. However, we found that most patients (85.1%) had never used CAM prior to TBI, which made the item “had ever used CAM” an irrelevant variable.

Questions on belief in CAM showed that more positive thoughts toward CAM resulted in a greater possibility that patients might choose CAM. Patients’ use of CAM is consistent with their attitudes toward CAM and health beliefs [22]. For instance, Chinese people generally regard Chinese medicine as being able to balance the qi of yin and yang. They also trust that it does not induce iatrogenic illness and nourishes the body with fewer side effects than conventional medicine does. Folk therapy is valued for its spiritual benefits, which relieve stress and enhance confidence [11,14]. CAM users also believe that CAM is holistic, natural, and associated with the spirit [23]. These varied beliefs affect the types and methods of medical treatment that patients use. In the view of patients, these different approaches to medicine are complementary, an attitude that reflects the concept of the yin-yang balance in Chinese culture. Our results show that most of the patients tended to use CAM and conventional medicine at the same time, suggesting that integrating traditional Chinese medicine and conventional medicine may become increasingly common in Taiwan’s health care system. Currently, in addressing the Taiwanese public’s preference for medical pluralism, some clinics integrated traditional Chinese and conventional medicine. We suggest that medical universities introduce scientifically approved CAM-related curricula, integrate CAM into programs that have previously used only conventional medicine, and provide opportunities for students to learn about complementary medical therapies.

This study had several limitations. Firstly, this study recruited only TBI patients who were scheduled for follow-up as outpatients; thus, information on those who did not need outpatient follow-up, those who lived outside the Taipei area and could not travel to the study hospital after discharge, and those who relied exclusively on CAM is limited, possibly causing a study sample bias. In addition, we used a convenience sample of patients attending an outpatient clinic who had received a single diagnosis at a single hospital. The limited sample size and homogeneity of patients, as well as the recalled bias of information in our study, limit the generalizability of the results. This study explored patients’ satisfaction with the efficacy of CAM based only on subjective perceptions and cannot provide evidence-based data on efficacy.

## Conclusion

Although the use of CAM is common for TBI patients receiving conventional medical health care in Taiwan, most patients did not inform health care personnel about their CAM use. TBI patients perceive combined use of CAM and conventional medicine as beneficial for their overall health.

## Competing interests

No conflict of interest has been declared by the authors.

## Authors’ contributions

BSG, SJH, and MFL conceived the idea for the study and all authors contribute to the design and concept. MFL and HLY carried out the data collection and statistical analysis. All authors read and approved the final manuscript.

## Pre-publication history

The pre-publication history for this paper can be accessed here:

http://www.biomedcentral.com/1472-6882/12/211/prepub
